# Wide-range and area-selective threshold voltage tunability in ultrathin indium oxide transistors

**DOI:** 10.1038/s41467-023-41041-y

**Published:** 2023-08-28

**Authors:** Robert Tseng, Sung-Tsun Wang, Tanveer Ahmed, Yi-Yu Pan, Shih-Chieh Chen, Che-Chi Shih, Wu-Wei Tsai, Hai-Ching Chen, Chi-Chung Kei, Tsung-Te Chou, Wen-Ching Hung, Jyh-Chen Chen, Yi-Hou Kuo, Chun-Liang Lin, Wei-Yen Woon, Szuya Sandy Liao, Der-Hsien Lien

**Affiliations:** 1 Institute of Electronics, National Yang Ming Chiao Tung University, Hsinchu, Taiwan; 2 Research & Development, Taiwan Semiconductor Manufacturing Company, Hsinchu, Taiwan; 3 Taiwan Instrument Research Institute, National Applied Research Laboratories, Hsinchu, Taiwan; 4 Department of Mechanical Engineering, National Central University, Jhongli City, Taiwan; 5 K-Jet Laser Tek Inc., Hsinchu, Taiwan; 6 Department of Electrophysics, National Yang Ming Chiao Tung University, Hsinchu, Taiwan

**Keywords:** Electrical and electronic engineering, Electronic devices

## Abstract

The scaling of transistors with thinner channel thicknesses has led to a surge in research on two-dimensional (2D) and quasi-2D semiconductors. However, modulating the threshold voltage (*V*_T_) in ultrathin transistors is challenging, as traditional doping methods are not readily applicable. In this work, we introduce a optical-thermal method, combining ultraviolet (UV) illumination and oxygen annealing, to achieve broad-range *V*_T_ tunability in ultrathin In_2_O_3_. This method can achieve both positive and negative *V*_T_ tuning and is reversible. The modulation of sheet carrier density, which corresponds to *V*_T_ shift, is comparable to that obtained using other doping and capacitive charging techniques in other ultrathin transistors, including 2D semiconductors. With the controllability of *V*_T_, we successfully demonstrate the realization of depletion-load inverter and multi-state logic devices, as well as wafer-scale *V*_T_ modulation via an automated laser system, showcasing its potential for low-power circuit design and non-von Neumann computing applications.

## Abstract (web summary)

The doping and threshold voltage modulation of 2D transistors remain challenging. Here, the authors report a wafer-scale optical-thermal method combining ultraviolet illumination and oxygen annealing to tune the threshold voltage of atomic-layer-deposited In_2_O_3_ transistors with 2 nm thickness.

## Introduction

The field-effect transistor (FET) is a fundamental component in modern electronics. The threshold voltage (*V*_T_) of a FET is a key parameter that determines its electronic functions, not only defining its switching modes but also scaling the supply voltage in the logic gates. In modern circuits, transistors are often tailored with different *V*_T_ during the manufacturing process to enhance performance and lower power consumption. The ability to precisely control the *V*_T_ of a transistor allows it to be used as a memory element, such as flash memory. In recent years, beyond-binary tunability between the “1” and “0” states has become particularly interesting in post–von Neumann applications, such as neuromorphic computation^1,2^, multi-state memory^3,4^ and multiplexed sensing^5^. The capacity to finely tune *V*_T_ over a wide range is crucial for these applications, as it directly impacts the storage capacity and weight precision of associated devices. It enables the development of advanced technologies that can perform complex tasks and have a wide range of applications in fields such as artificial intelligence, machine learning, and the Internet of Things (IoT).

Atomically thin semiconductors, including two-dimensional and other quasi-2D materials, have shown great potential to develop beyond-silicon electronics. Indium oxide (In_2_O_3_) recently emerged as a promising channel material for FETs as it can be thinned down to 1 nm and maintains high electron mobility beyond 100 cm^2^ V^–1^ s^–1^, showing high on-state drain current density *I*_D_ > 20 A mm^−1^ (refs. ^6,7^). This advancement allows for the scaling of In_2_O_3_ to align with modern technology nodes, and is expected to complement silicon-based systems for future back-end-of-line (BEOL) integration, expanding its range of applications beyond display technology. Moreover, In_2_O_3_ exhibits high potential to engineer the *V*_T_ as a benefit generally granted to the oxide semiconductors (OS)^8^. In modern FETs, *V*_T_ is tuned during fabrication by adjusting the implanted dopant concentration or by modifying the work function of the gate. To enable dynamic control over *V*_T_, additional charge-trapped layers are required, e.g., a floating gate^9,10^. In OS-based FETs, *V*_T_ can be tuned by various approaches such as post-fabrication thermal annealing^11,12^, chemical doping^13^, passivation^14^, metal decorations^15^, and incorporating a gate electrode with selected work functions^16^, etc. It is known that vacancy-related surface effects play a crucial role in altering the charge carrier density of the OS, leading to the ease of *V*_T_ tunability. Even though the vacancy-related surface effects can be easily controlled, scalable *V*_T_ modulation with fine control over wide ranges is still challenging.

In this work, we report wide-range *V*_T_ tunability in ultrathin In_2_O_3_ FETs achieved via a simple optical-thermal combined method. The method involves alternating ultraviolet (UV) illumination and oxygen annealing to achieve negative and positive *V*_T_ tuning, respectively. The *V*_T_ of a 2 nm-thick In_2_O_3_ transistor (30 nm-thick SiO_2_ as the dielectric) exhibits a tunable window of 20 V with a resolution of 0.05 V, equivalent to a maximum change of sheet carrier density (*n*_2D_) from 2$\times$10^10^ cm^−2^ to 2$\times$10^12^ cm^−2^ with a resolution of 10^9^ cm^−2^. Importantly, this method is an entire post-fabrication process, and the *V*_T_ modulation is reversible. We show that the *V*_T_ of distributed transistors in a circuit can be selectively tuned, enhancing the gain of a depletion-load inverter by an order of magnitude. Exploiting the spatial *V*_T_ tunability, we show that such local control of *V*_T_ can be used to pattern the *V*_T_ profile in a channel of a transistor to enable multi-step transfer characteristics, advocating potential for innovative logic design and neuromorphic applications. To further demonstrate the wafer-scale practicality, we fabricated In_2_O_3_ transistors on a 4-inch wafer and utilize the industry-level laser illumination system to achieve automatic large-area *V*_T_ tuning for selected In_2_O_3_ transistors across the entire wafer.

## Results and discussion

**Fig. 1:**
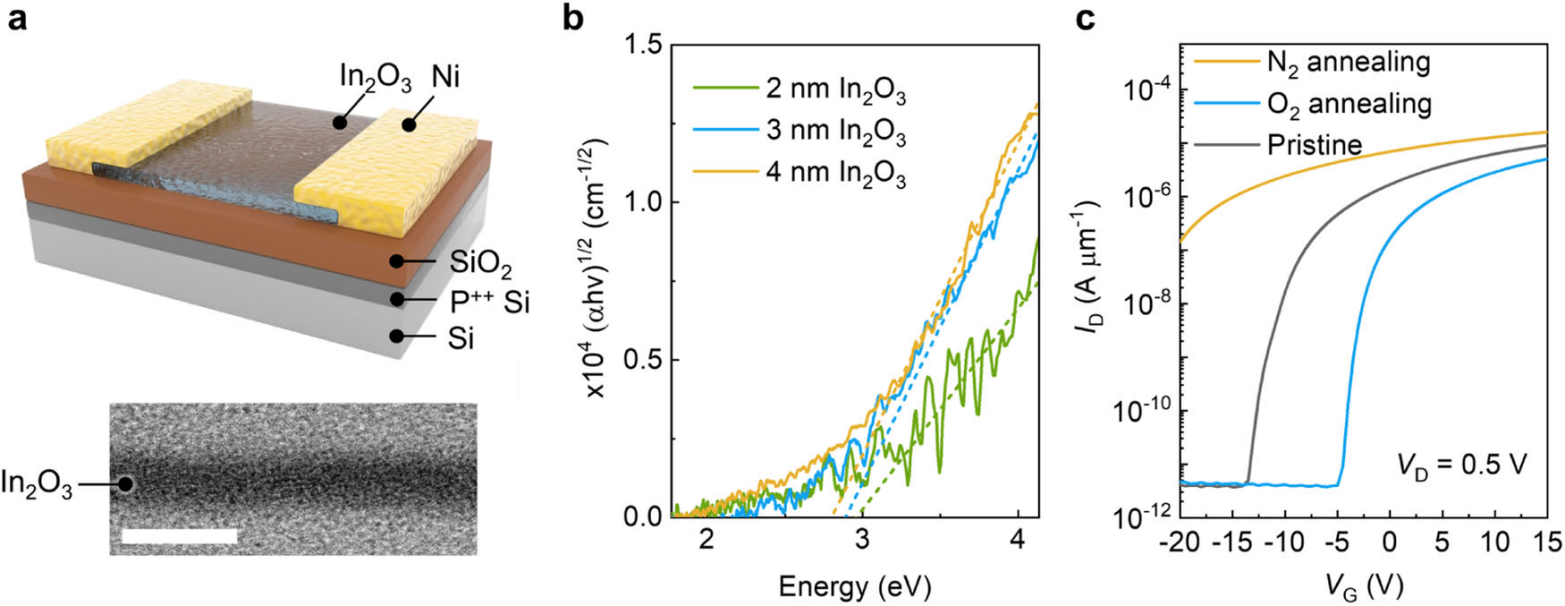
Device structures and high-resolution transmission electron microscopy (HRTEM) images of atomic layer deposition (ALD) deposited In_2_O_3_ transistors. **a** Schematic of a 2 nm In_2_O_3_ transistor. The inset shows the HRTEM image of the ALD-deposited ultrathin In_2_O_3_ films. The scale bar is 5 nm. **b** Tauc plot of the In_2_O_3_ films as a function of film thickness. The fitting of the curve is done based on the Tauc model (α: absorption coefficient; h: Planck’s constant; ν: frequency of vibration). **c** Transfer curves of 2 nm In_2_O_3_ transistors with channel width/length of 10/2 µm and annealed under N_2_ and O_2_ for 30 min at 150 °C. Note that the amounts of threshold voltage (*V*_T_) shifts are saturated after 30 minutes (*I*_D_: drain current; *V*_D_: drain voltage; *V*_G_: gate voltage).

Indium has been utilized across a variety of applications (element scarcity is shown in Supplementary Table 1), including displays (e.g., IGZO), transparent electrodes (e.g., ITO), and solar cells (e.g., CIGS). In this study, ultrathin In_2_O_3_ is employed as the channel material of a transistor, as shown schematically in Fig. 1a. The transistor is fabricated using atomic layer deposition (ALD) to form a 2–4 nm In_2_O_3_ layer on p^++^Si substrates with a 30 nm-thick SiO_2_. Nickel is used as the source and drain contacts. It is important to note that the entire fabrication process is compatible with back-end-of-line (BEOL) CMOS processes, as it is carried out below the thermal budget of 300 °C (“Methods”)^17^. The high-resolution transmission electron microscopy (HRTEM; Supplementary Fig. 1) image reveals the amorphous nature and the atomic level uniformity of the In_2_O_3_ films is confirmed by AFM (Supplementary Fig. 2), which effectively accounts for the minimal device-to-device variation (Supplementary Fig. 3). It has been shown that In_2_O_3_ shows high electron mobilities even in an amorphous phase^18^. This is because the electronic property of OS is insensitive to crystallinity. Their transport is determined by the overlapping of s orbitals between neighboring metal atoms (e.g., indium) and is less affected by structural disorders^8^. The Tauc plot extracted from absorption spectra (Fig. 1b and Supplementary Fig. 4) shows that the bandgap of In_2_O_3_ increases with decreasing channel thickness (*t*_ch_), from 2.7 eV (*t*_ch_ = 4 nm) to 3 eV (*t*_ch_ = 2 nm) due to the enhanced quantum confinement effect. This finding is similar to the layer-dependent bandgap changes observed in 2D materials, revealing the quasi-2D feature of the ultrathin In_2_O_3_ films^19^.

**Fig. 2:**
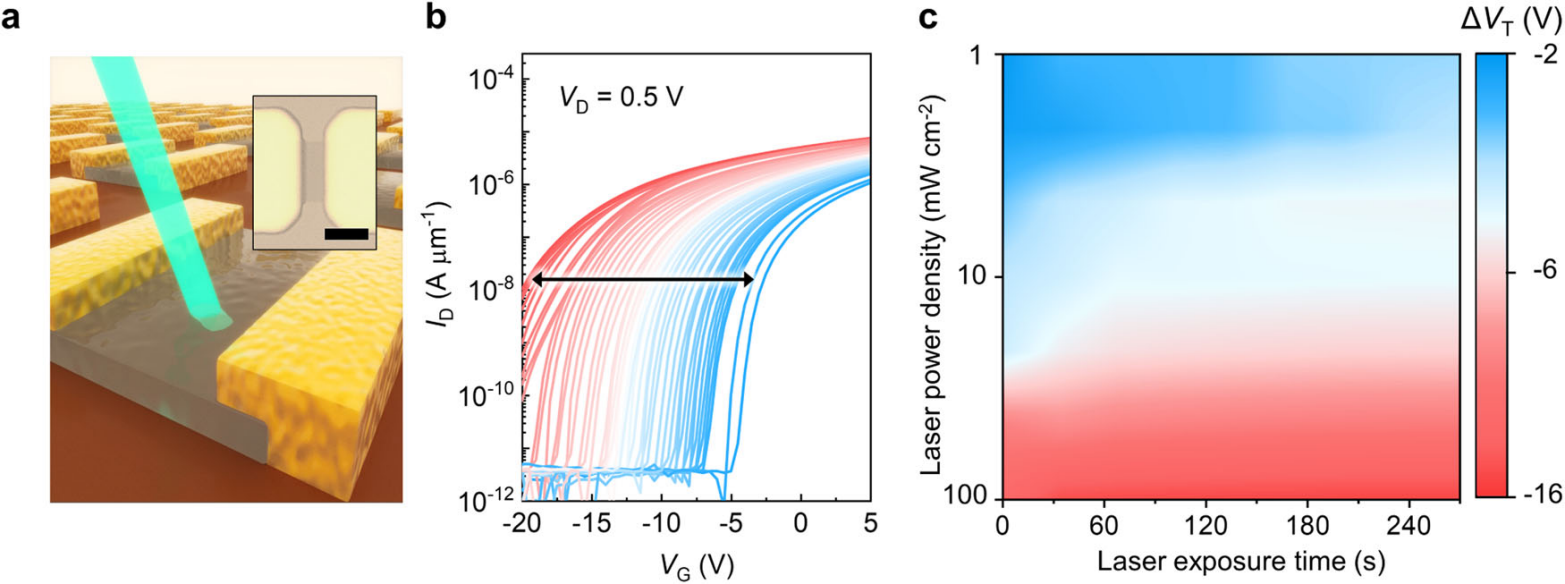
*V*_T_ tuning in ultrathin In_2_O_3_ transistors via ultraviolet (UV) exposure. **a** A schematic of *V*_T_ tuning in ultrathin In_2_O_3_ transistors through UV exposure combined with thermal annealing; the inset is the microscopic image of the transistor with a scale bar of 5 µm. **b** Transfer curves of 2 nm In_2_O_3_ transistors with channel width/length of 10/2 µm after post treatments. The arrow represents the transition of transfer curves with UV light exposure (red lines) and O_2_ annealing (blue lines) (*I*_D_: drain current; *V*_D_: drain voltage; *V*_G_: gate voltage). **c** A contour plot of *V*_T_ shifts as a function of UV exposure time and power density. Devices were annealed at 150 °C in O_2_ for 30 min to reset the *V*_T_ before each UV exposure measurement. The absorbed power density increasing from 1$\times$10^6^ mW cm^−2^ to 1$\times$10^8^ mW cm^−2^ for exposure times from 30 s to 300 s under 365 nm laser illumination. The measurement time interval is 30 s and the transfer characteristics of the device are immediately measured (<5 s) after UV illumination. The plot consists of 7 different power densities and 10 different exposure times.

**Fig. 3:**
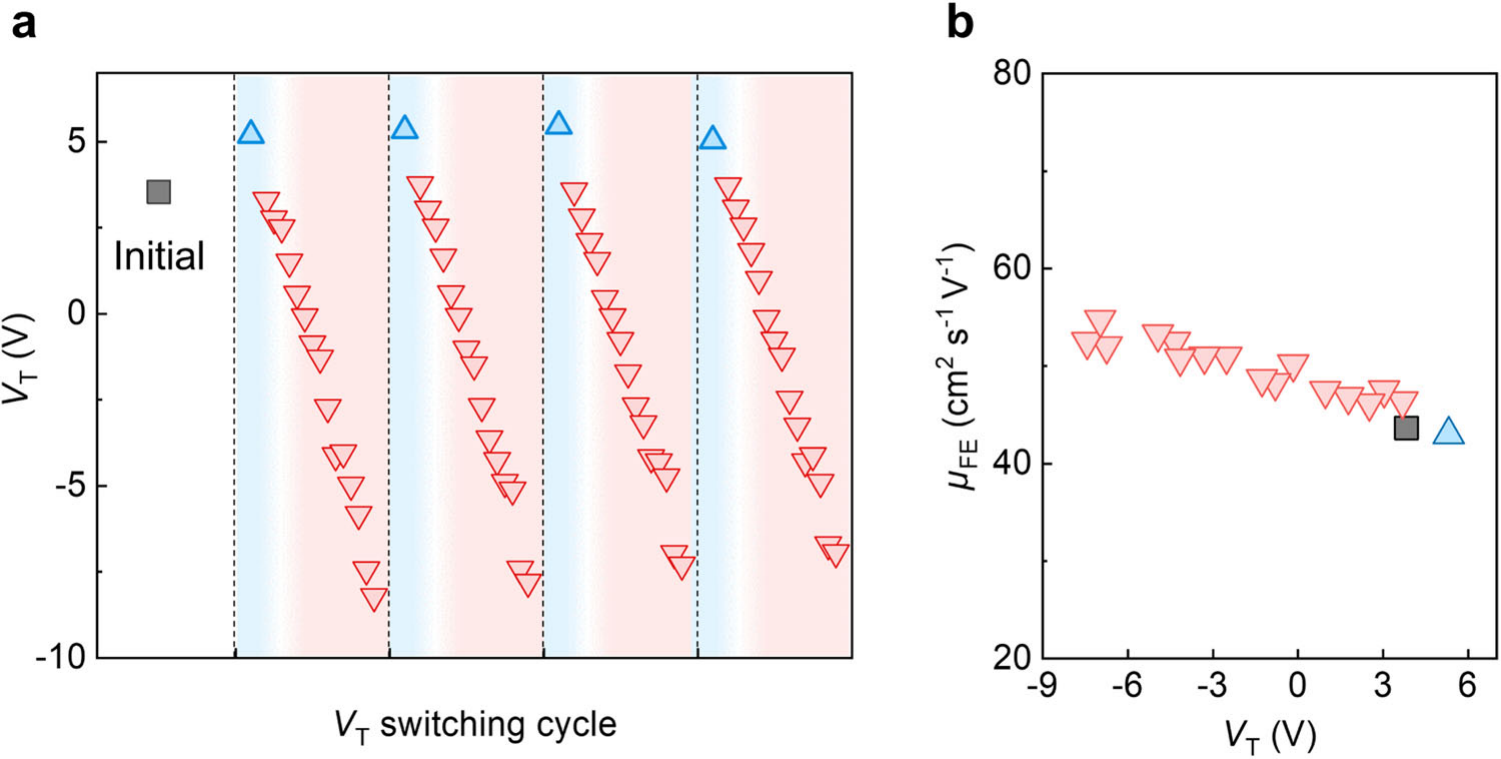
Reversibility of *V*_T_ tuning in ultrathin In_2_O_3_ transistors. **a** *V*_T_ of a 2 nm In_2_O_3_ transistor with channel width/length of 10/2 µm during multiple O_2_ annealing and UV exposure cycles. In_2_O_3_ transistors are thermally annealed under O_2_ for 30 min, followed by UV illumination under a power density of 1 mW cm^−2^. The blue triangles are the *V*_T_ after O_2_ annealing. The red triangles are the *V*_T_ after UV exposure. The dashed lines separate different switching cycle and the shaded areas represent the treatments (blue: O_2_ annealing; red: UV exposure). **b** The field effect mobility (${\mu }_{{FE}}$) of ultrathin In_2_O_3_ transistors as a function of *V*_T_ tuned by the proposed method.

Figure 1c shows the transfer characteristics (*I*_D_–*V*_G_) of 2 nm In_2_O_3_ transistors annealed at 200 °C in O_2_ and N_2_ environments. The transfer characteristics (*I*_D_–*V*_G_) of In_2_O_3_ below 2 nm is shown in Supplementary Fig. 5 and saturation behavior (*I*_D_–*V*_D_) is shown in Supplementary Fig. 6. O_2_ and N_2_ annealing lead to *V*_T_ shifts to +6 V and −20 V, respectively; both show effective tuning of *V*_T_ after 30 min of annealing (the *V*_T_ extraction method is described in the “Methods” section). While the traditional annealing method can effectively tune the *V*_T_ of In_2_O_3_ transistors, precise control of *V*_T_ over the tuning range is difficult. Figure 2a illustrates the proposed approach to achieve fine-tuning of *V*_T_ in ultrathin In_2_O_3_ transistors. The device is first subjected to O_2_ annealing, which shifts the *V*_T_ to +6 V. Subsequent UV irradiation (365 nm) on In_2_O_3_ transistors shifts the *V*_T_ to the negative values (Fig. 2b), where the magnitude of *V*_T_ shifts depends on the exposure time, incident power density and wavelength (Fig. 2c and Supplementary Figs. 7 and 8). Note that UV exposure causes an accumulation of *V*_T_ shift, leading to a saturation point of −15 V with a tuning resolution of 0.05 V within a tunable window Δ*V*_T_ of 21 V (Supplementary Fig. 9). To verify reproducibility of our approach, we tested devices with identical fabrication processes but different metal contacts (Pd, Pt) and substrates (10 nm HfO_2_ as the gate dielectric). All results exhibited consistent trends, regardless of substrate and metal contacts. (Supplementary Fig. 10). Importantly, the *V*_T_ modulation using the proposed approach is reversible. UV-exposed In_2_O_3_ transistors with negative *V*_T_ can be reset to have a value of 5 V after O_2_ annealing. Figure 3a shows repeated *V*_T_ modulation achieved by multiple O_2_ annealing-UV exposure cycles. The field effect mobility (${\mu }_{{FE}}$) of the transistors is maintained at 49 ± 5 cm^2^ V^–1^ s^–1^, indicating that the properties of the In_2_O_3_ transistors are not significantly affected by the tuning *V*_T_ processes (Fig. 3b). Additionally, we also conduct the bias stress experiment to investigate behavior and reliability of transistors under different bias conditions after the treatment of O_2_ annealing and UV exposure (Supplementary Fig. 11). A gate voltage was applied at ±15 V for 1000 s, with both source and drain grounded. The transfer characteristics of the device are immediately measured (<1 s) after bias stress. Furthermore, to evaluate the potential damage caused by laser exposure, we performed finite element simulations to analyze the temperature changes. The results indicate only a slight increase in temperature at the applied incident power densities (Supplementary Fig. 12).

**Fig. 4:**
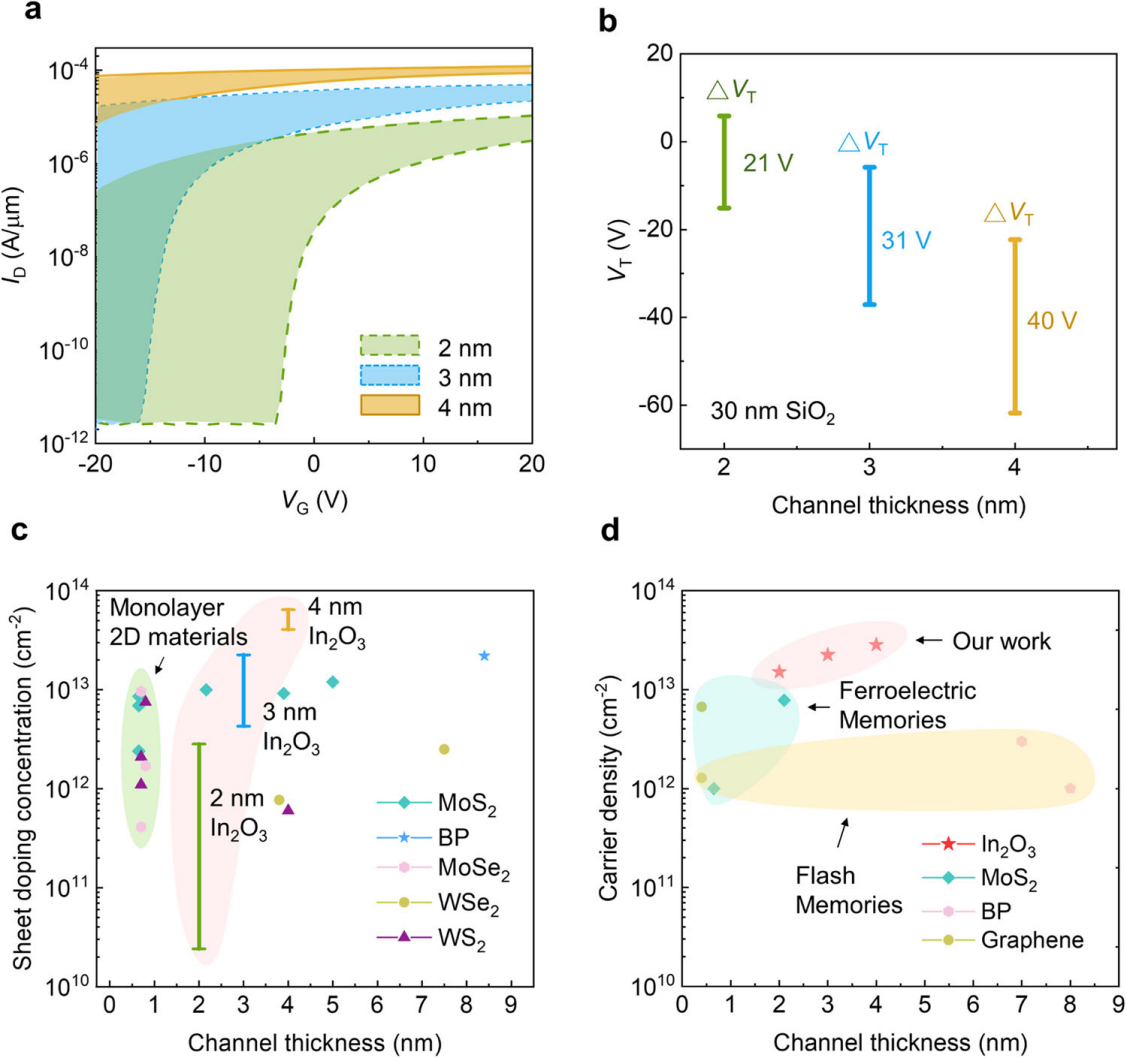
Tunable *V*_T_ windows and benchmarks of In_2_O_3_ transistors versus other ultrathin transistors. **a** The range of the tunable window of In_2_O_3_ transistors with various In_2_O_3_ thicknesses, channel width/length of 10/2 µm. **b** The range of *V*_T_ after treatment. **c** Benchmark of sheet carrier density (*n*_2D_) for different classes of ultrathin semiconductors with different thicknesses^20–30^, calculated using both the Drude model and the parallel-plate capacitor model. The shaded areas represent different classes of materials (red: In_2_O_3_; purple: monolayer 2D materials). **d** The Δ*n*_2D_ for different classes of ultrathin semiconductors based on capacitively charged structures. Shaded area represents different material categories (red: In_2_O_3_; green: ferroelectric memories; yellow: flash memories).

The size of the tunable window Δ*V*_T_ is dependent on the thickness of the In_2_O_3_, as shown in Fig. 4a, b (*V*_T_ shift as a function of channel length is shown in Supplementary Fig. 13). In_2_O_3_ with a thickness of 4 nm shows the largest tunable window, whereas In_2_O_3_ with a thickness of 2 nm is capable of being tuned between the enhancement mode (*V*_T_ > 0 V) and the depletion mode (*V*_T_ < 0 V). The offsets of tunable window for different thickness can be explained by the quantum confinement effect on the trap-neutral level model^19^. Fermi level is located deeply inside the conduction band for thicker In_2_O_3_ and aligns within the bandgap for thinner In_2_O_3_ as the bandgap is enlarged due to the enhanced quantum confinement effect.

To further quantify the *V*_T_ shift, we extract 2D carrier density (${n}_{2D}$) using the Drude model ${n}_{2D}={I}_{D}L/({qW}{V}_{D}{\mu }_{{FE}})$, where *q* is the electron charge, *I*_D_ is the source-drain current at zero gate voltage, *V*_D_ is the source-drain voltage, and *μ* is the field effect carrier mobility. Based on the Drude model, we benchmark our method against other *V*_T_ tuning methods used in different classes of ultrathin semiconductors. Figure 4c shows the *n*_2D_ of 2D semiconductors modulated by various chemical doping schemes^20–30^, which ranges from 10^12^ to 10^13^ cm^−2^ depending on the doping methods and materials (details in Supplementary Table 2). The tunable ranges of *n*_2D_ achieved in In_2_O_3_ transistors vary from 10^10^ cm^−2^ to 10^13^ cm^−2^ depending on the thickness. 2 nm In_2_O_3_ exhibits the widest *n*_2D_ tunable window (2$\times$10^10^ cm^−2^ to 2$\times$10^12^ cm^−2^), indicating the proposed method is competitive among various 2D doping techniques. The proposed methods offer a comparable effect to chemical doping, while providing the advantages of reversibility and area selectivity. In contrast to the substantial challenges associated with chemical doping in 2D materials, our method showcases the ease of achieving effective carrier concentration tuning in ultrathin In_2_O_3_. Moreover, our approach minimizes fabrication processes, thereby enhancing its potential for seamless integration in BEOL applications.

Another commonly used approach to control the *V*_T_ of a transistor is through device design, usually done by inserting a layer that can be capacitively charged. For instance, flash memories store charge in a charge-trapping layer to modulate *V*_T_ through the trapping and de-trapping process. Similarly, ferroelectrics modulate *V*_T_ by controlling the polarization of a ferroelectric layer. The density of stored charge carriers *n*_2D_ is around 10^12^ cm^−2^ to 10^13^ cm^−2^ for graphene and related 2D materials-based flash memories and ferroelectric memories. The Δ*n*_2D_ for In_2_O_3_ extracted by the parallel-plate capacitor model (1.5$\times$10^13^, 2.3$\times$10^13^ and 2.8$\times$10^13^ cm^−2^ for 2, 3 and 4 nm, respectively) is of the same order as flash memories and ferroelectric memories without extra gates (Fig. 4d)^9,31–35^. The details of 2D-based charge-storing schemes are summarized in Supplementary Table 3. Compared to other 2D doping and capacitively charged techniques, the proposed method demonstrates a more effective way to largely tune the carrier density in In_2_O_3_ transistors, enabling In_2_O_3_ transistors for innovative circuit and memory applications.

We conducted cyclically annealing between N_2_ and O_2_ environments to investigate the mechanism (Supplementary Fig. 14). The results demonstrate that the *V*_T_ shift by N_2_ and O_2_ annealing is reversible, similar to the reversibility observed in UV exposure and O_2_ annealing processes. This result implies that the mechanisms underlying these two approaches may be identical. It is known that electronic properties of amorphous In_2_O_3_ can be altered by oxygen-related defects such as oxygen vacancies and oxygen adatoms on the surface^36^. Oxygen vacancies act as shallow donors and contribute to the spontaneous n-type conductivity of the In_2_O_3_ (the shallow donor level is observed in scanning tunneling microscopy (STS) shown in Supplementary Fig. 15). Oxygen adatoms, on the other hand, act as acceptor-like traps that counter-dope the n-type OS. Annealing in oxygen-rich or oxygen-scarce (N_2_ or vacuum) environments causes a rebalance of physically adsorbed oxygen adatoms, leading to positive and negative *V*_T_ shifts, respectively. Furthermore, exposure to UV light generates holes that neutralize the negatively charged oxygen adatoms, resulting in their detachment and a decrease in the *V*_T_. This proposed mechanism is supported by the measurement of *V*_T_ retention in different atmospheres, as shown in Supplementary Fig. 16. Additionally, the *V*_T_ exhibits almost no change over time in an ultra-high vacuum environment (10^−9^ torr), which further highlights the crucial role of oxygen adatoms in this process. The results suggest that the stability could be improved by implementing effective isolation or passivation to minimize the exposure of In_2_O_3_ to the atmosphere.

### Demonstration of depletion-load inverter and multi-step logic

**Fig. 5:**
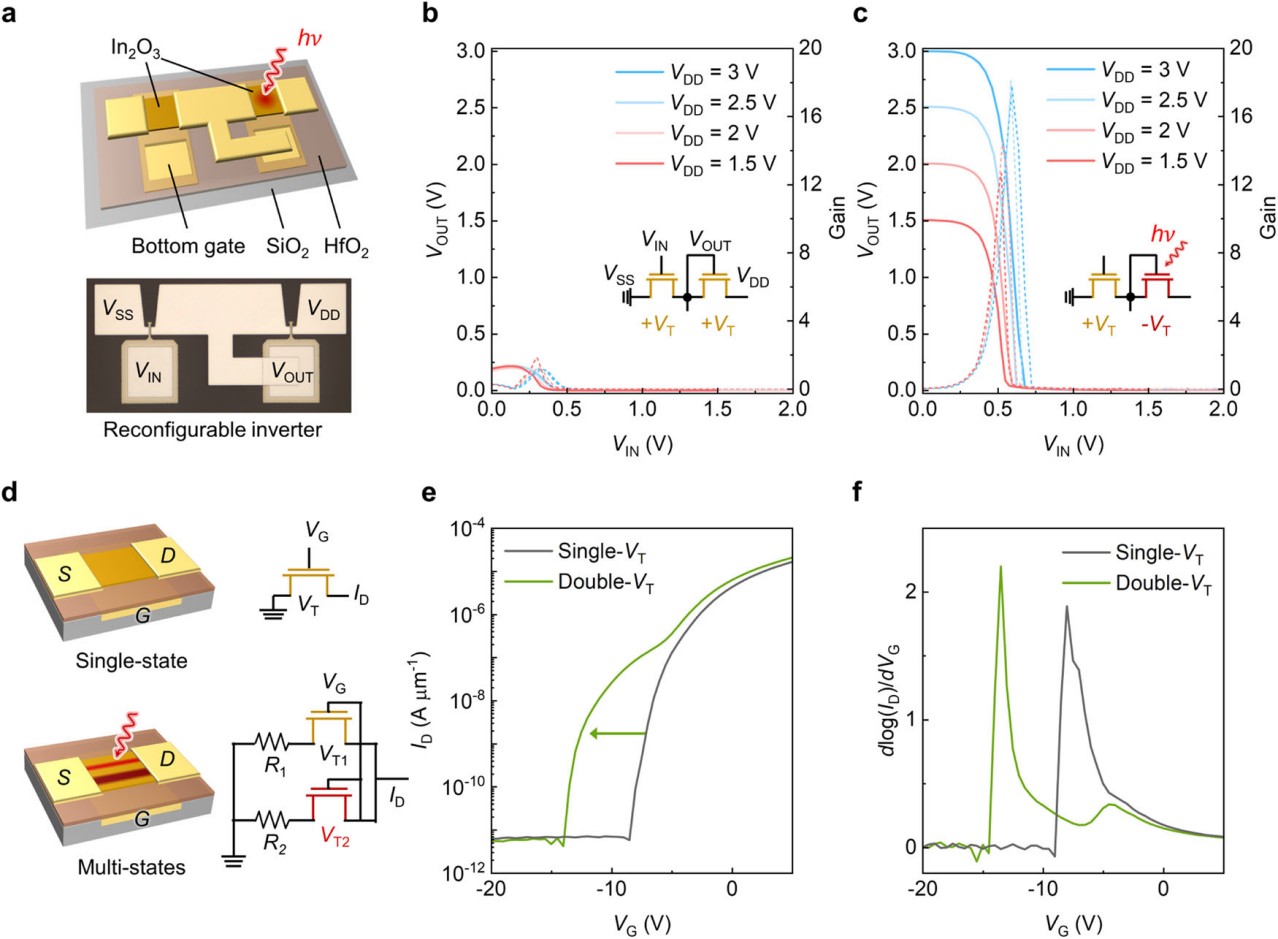
Applications of ultrathin In_2_O_3_ transistor with laser exposure. **a** A schematic and optical microscope image of In_2_O_3_ inverter (*h*: Planck’s constant; *ν*: frequency of vibration; *V*_DD_: drain voltage; *V*_SS_: source voltage; *V*_IN_: input voltage; *V*_OUT_: output voltage). **b** The voltage transfer characteristics of 2 nm In_2_O_3_ inverter with channel width/length of 10/2 µm before UV laser illumination. **c** The voltage transfer characteristics of In_2_O_3_ inverter of 2 nm In_2_O_3_ inverter with channel width/length of 10/2 µm after UV laser illumination. **d** A schematic of ultrathin In_2_O_3_ transistor with multi-state logic function (*V*_G_: gate voltage; *I*_D_: drain current; *R*_1_: resistance of first channel; *R*_2_: resistance of second channel; *V*_T1_: threshold voltage of first channel; *V*_T2_: threshold voltage of second channel). **e**, The transfer characteristics of In_2_O_3_ transistor with UV laser illumination on the specific area. **f** The transfer characteristics with the first-order derivative of log(*I*_D_) from **e**.

The proposed method allows for precise control of *V*_T_, making it useful for advanced low-power circuits^37^. Here, we demonstrate a depletion-load inverter by adjusting the *V*_T_ of the selected In_2_O_3_ transistors in a circuit, achieved by employing a micro-laser system that can focus UV exposure selectively on In_2_O_3_ transistors (details in “Methods”). Figure 5a illustrates the layout of an inverter circuit with two n-type In_2_O_3_ transistors connected by local bottom gates. Both transistors are set to enhancement mode via O_2_ annealing, resulting in a *V*_T_ shift to +5 V. When *V*_DD_ is applied, *V*_OUT_ is limited as the channel resistance of the load transistor is large, resulting in a small gain of 2 for *V*_DD_ = 3 V (Fig. 5b). After exposing the load transistor to UV light, tuning the selected transistor to depletion mode, the inverter becomes a depletion-load inverter with an enhanced gain of 18 (Fig. 5c). Note that the depletion load inverter serves as a proof of concept for our proposed technology. Integrating a complementary MOS configuration has the potential to further optimize the performance of inverters, resulting in reduced power consumption and improved spatial advantages. The functionality of the circuit is activated via the proposed method with local *V*_T_ tunability, giving a higher degree of freedom to design and calibrate the circuit even after fabrication.

The ability to locally adjust the *V*_T_ with high spatial resolution in In_2_O_3_ transistors allows for creating non-uniform *V*_T_ patterns in the channel and enables new functions. We demonstrate that a reconfigurable In_2_O_3_ transistor can switch between binary and multi-state logic without the need for multiple gates^38^ or heterojunctions^39^. Figure 5d illustrates a transistor with uniform *V*_T_ along the channel detailed in Supplementary Fig. 17. After UV exposure on the selected area, non-uniform *V*_T_ pattern forms on the channel. The equivalent circuit configuration changes depending on the scanning pathways, consisting of parallel resistors and transistors with different *V*_T_ depending on the exposure time. As a result, a single swing in transfer characteristics turns to double-swing characteristics (Fig. 5e, f), increasing the information capacity of a single transistor. The demonstration of an In_2_O_3_-based multi-state logic device introduces an alternative strategy to design reconfigurable multifunctional logic circuits and has the potential for neuromorphic applications (Supplementary Fig. 18)^40,41^.

### Wafer-scale *V*_T_ modulation

**Fig. 6:**
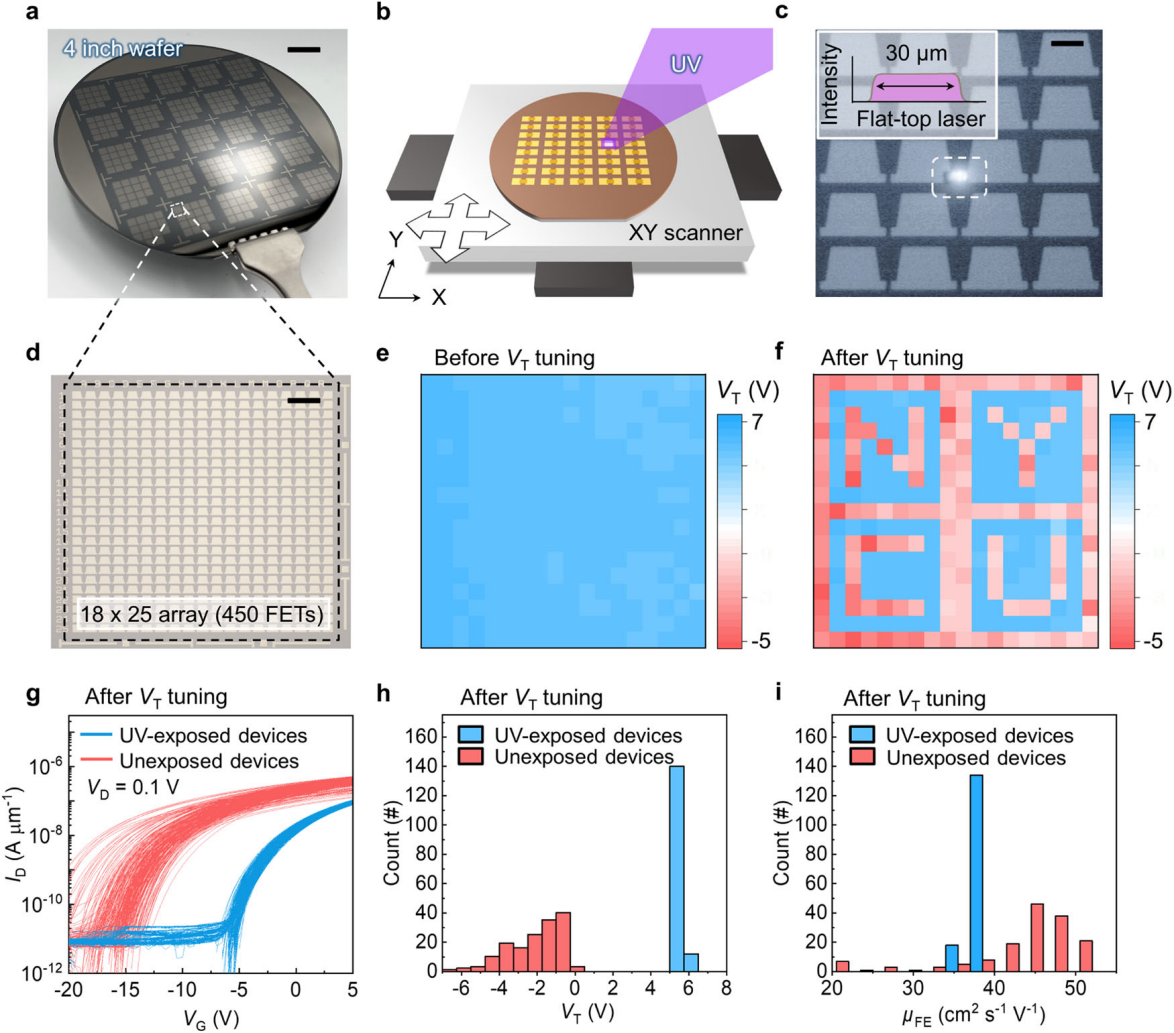
Large-area *V*_T_ modulation. **a** An image of ultrathin In_2_O_3_ transistor arrays on a 4-inch wafer. The scale bar is 1 cm. **b** Schematics of the automated laser illumination system used for *V*_T_ modulation. **c** An optical microscope image of UV laser exposure on the designated channel area. The inset is the illustration of flat-top laser beams. The scale bar is 50 µm. **d** An optical microscope image of the basic array unit on 4-inch wafer consisted of an 18$\,\times \,$25 array (450 transistors**)**. The scale bar is 200 µm. **e** A contour plot showing the *V*_T_ of the transistors in the array before *V*_T_ tuning. **f** A contour plot showing the *V*_T_ of the transistors in the array after *V*_T_ tuning. **g** The transfer curves of the devices with channel width/length of 10/2 µm in the array after *V*_T_ tuning (red line: UV exposed areas; blue line: unexposed area). **h** The histogram of *V*_T_ of the devices in the array after *V*_T_ tuning. **i** The histogram of ${\mu }_{{FE}}$ of the devices in the array after *V*_T_ tuning.

To further demonstrate the practicability of our technique, we fabricated In_2_O_3_ transistors on a 4-inch wafer (Fig. 6a) and employed an automated laser illumination system (Laser Lift-Off System; K-JET LASER TEK Inc.) for large-scale *V*_T_ tuning (Fig. 6b). The system allows for precise alignment to direct focused flat-top illumination onto selected devices, enabling localized *V*_T_ modulation (Fig. 6c, Supplementary Movie 1 and tool details in Methods). We demonstrate the scalability by tuning *V*_T_ for 450 transistors in an 18 $\times$ 25 array (Fig. 6d). The initial *V*_T_ was set to ~7 V through O_2_ annealing for all transistors (Fig. 6e), and the *V*_T_ of the laser-exposed transistors were tuned to negative values (Fig. 6f). The transfer curves after *V*_T_ modulation were shown in Fig. 6g, demonstrating the consistency of the proposed approach. The *V*_T_ of unexposed transistors remained the same after the large-area tuning, as shown in Fig. 6h. The ${\mu }_{{FE}}$ distribution shows that the mobility increases slightly after UV exposure (Fig. 6i), in agreement with previous results. Note that the device-to-device variation, induced by the variation in film quality across the wafer, may potentially be mitigated through the utilization of industrial-level ALD tools. Furthermore, the *V*_T_ tuning precision could be improved by the incorporation of patterned photomasks. This demonstration not only shows capability of wafer-scale fabrication and *V*_T_ tuning on multiple transistors, but also opens up possibilities for enhancing circuit functionality beyond *V*_T_ adjustment.

In summary, we demonstrate the wide-range *V*_T_ tunability in ultrathin In_2_O_3_ using an optical–thermal method. This approach is comparable to doping and capacitively charged techniques used in other ultrathin semiconductors and avoids the need for external dopants and complex processes. Our demonstration of a depletion-load inverter suggests that this *V*_T_ tuning method has the potential to enable new functionalities in combinational logic circuits. Additionally, we have demonstrated a single device with multi-state logic function, which opens up new possibilities for the design of multifunctional logic circuits and non-von Neumann computing. Moreover, we conducted large-scale *V*_T_ modulation using an industry-level tool to highlight the practical applicability of this method alongside individual device testing.

## Methods

### Device fabrication

The device fabrication started with a standard wet and dry pre-clean of 300 mm Si substrate. The highly phosphorus-doped Si, which carrier density is over 10^21^ cm^−3^, was grown on Si substrate as a global back-gate. Then, 30 nm SiO_2_ was deposited by ALD at 260 °C with (H_2_Si[N(C_2_H_5_)_2_]_2_) as the precursor. Afterwards, In_2_O_3_ thin films with different thicknesses were deposited by ALD at 200 °C using (CH_3_)_3_In (TMIn) and O_3_ as indium (In) and oxygen (O) precursors. The active areas of In_2_O_3_ were defined by lithography with HCl etching with the channel width/length of 10/2 µm. Standard lithography patterning and lift-off procedures were performed to contact the In_2_O_3_ thin films with metal electrodes. 40 nm Ni was deposited by e-beam evaporation on In_2_O_3_ to serve as source/drain ohmic contacts.

### Inverter fabrication

The inverter fabrication started with a standard wet and dry pre-clean of 250 mm Si with a 50 nm SiO_2_ substrate. Then, the local back gate of 40 nm Ni was deposited by e-beam evaporation. Afterwards, 6 nm HfO_2_ was deposited by ALD at 250 °C with TDMAHf as a precursor for 70 cycles. After the gate dielectric deposition, 2 nm In_2_O_3_ thin films were deposited by ALD at 200 °C using (CH_3_)_3_In (TMIn) and O_3_ as In and O precursors. The active areas of In_2_O_3_ were defined by lithography with HCl etching for 5 s. Then, the contact holes were patterned by standard lithography, followed by BOE etching for 5 min. Finally, a Ni (40 nm) film was deposited by e-beam evaporation to serve as source/drain ohmic contacts and metal line interconnections.

### Device characterization

As-grown In_2_O_3_ was then measured for thickness using a transmission electron microscope (TEM). A focused ion beam (FIB) system (Auriga, Carl Zeiss) was used to fabricate the cross-sectional specimen, which was then examined by a TEM (none-Cs Metrios). The electronic characteristics were measured by an Agilent B2902B source. The ${\mu }_{{FE}}$ and *V*_T_ of ultrathin In_2_O_3_ transistors were determined in linear regime using conventional MOSFET equation for ${V}_{D}\ll {V}_{G}-{V}_{T}$: ${I}_{D}=\frac{W}{L}{\mu }_{{FE}}{C}_{{OX}}({V}_{G}-{V}_{T}){V}_{D}$, where *C*_OX_ is the oxide capacitance. The result of ${\mu }_{{FE}}$ can be obtained from ${\mu }_{{FE}}=\frac{L{g}_{m}}{W{C}_{{OX}}{V}_{D}}$, where *g*_m_ is maximum transconductance. The resulted *V*_T_ was determined by linear extrapolation, which was conducted by plotting *I*_D_ versus *V*_G_, extrapolating from maximum transconductance (*g*_m_) to *I*_D_ = 0 and adding *V*_D_/2 to obtain the intercept at *V*_G_ axis.

### Laser exposure

The 365 and 532 nm laser beams were generated by diode lasers (RGB Laser systems, Lambda Beam). The laser light passes through a shutter (NM Laser Product, LST-5VDC) to control the laser exposure time. Following the shutter, an ND filter was used to control the laser incident power. After that, the laser beam (365, 532 nm) was focused on the device through a fixed optical path using a ×50 objective lens (OLYMPUS, LMPlanFL N ×50, NA  = 0.5) with a laser spot diameter of ~10 μm. Meanwhile, the absorbed power was measured by a power meter which comprised a detector (Thorlab, S120VC) and a meter (Thorlab, PM100D).

### Thermal annealing

The fabricated devices were placed in a customized chamber with two gas inlets. Then, the devices were annealed in O_2_ and N_2_ with 1 liter per minute gas flow for 30 min at 150 °C. The pressure was kept at about 1 atm.

### Absorption spectra

The In_2_O_3_ films were deposited on a 200 μm-thick glass substrate following the same ALD process as mentioned before. An ellipsometer determined the thickness of In_2_O_3_ films for absorption measurement. A Hitachi U-4100 spectrophotometer was used to investigate the transmittance of samples between the 300 and 700 nm range with an integrating sphere. Absorptance of the films was calculated using the relation *A* = 100 − (*T* + *R*).

### Wafer-scale *V*_T_ modulation

Wafer-scale *V*_T_ modulation was achieved using an automatic laser illumination tool (Laser Lift-Off System; K-JET LASER TEK Inc.). The laser spot size and shape can be customized by a mask based on our specific needs, allowing for arbitrary adjustments. The laser intensity is uniformly distributed across the entire exposure area (20 µm $\times$ 1 µm for this study), commonly known as flat-top beams, achieved via applying a mask on the optical path to ensure consistent laser exposure within the targeted region. Laser pulses with energy of 0.0015 J/cm^2^, a wavelength of 365 nm, and a pulse width of 26 ns were directed onto the desired regions or devices. The scanning rate is ~1 ms/transistor.

### Supplementary information

Supplementary Information

Peer review file

Description of Additional Supplementary Files

Supplementary Movie 1

## Data Availability

Relevant data supporting the key findings of this study are available within the article and the Supplementary Information file. All raw data generated during the current study are available from the corresponding authors upon request.
