# Supplementary material for: Wide-range and area-selective threshold voltage tunability in ultrathin indium oxide transistors
Source: Nat Commun. 2023 Aug 28;14:5243. doi: 10.1038/s41467-023-41041-y (PMC10462674; doi:10.1038/s41467-023-41041-y)
Supplement: Supplementary file 1 — Supplementary Information [file 41467_2023_41041_MOESM1_ESM.pdf]

Supplementary Information

Wide-range and area-selective threshold voltage tunability in ultrathin indium oxide transistors

Robert Tseng[1], Sung-Tsun Wang[1], Tanveer Ahmed[1], Yi-Yu Pan[1], Shih-Chieh Chen[1] Che-Chi Shih[2], Wu-Wei Tsai[2], Hai-Ching Chen[2], Chi-Chung Kei[3], Tsung-Te Chou[3], Wen-Ching Hung[4,5], Jyh-Chen Chen[4], Yi-Hou Kuo[6], Chun-Liang Lin[6], Wei-Yen Woon[2], Szuya Sandy Liao[2], Der-Hsien Lien[1]**

[1]Institute of Electronics, National Yang Ming Chiao Tung University, Hsinchu, Taiwan

[2]Research & Development, Taiwan Semiconductor Manufacturing Company, Hsinchu, Taiwan

[3]Taiwan Instrument Research Institute, National Applied Research Laboratories, Hsinchu, Taiwan

[4]Department of Mechanical Engineering, National Central University, Jhongli City, Taiwan

[5]K-Jet Laser Tek Inc., Hsinchu, Taiwan

[6]Department of Electrophysics, National Yang Ming Chiao Tung University, Hsinchu, Taiwan

*Address correspondence to: wywoona@tsmc.com, dhlien@nycu.edu.tw

This supplementary information file includes:

Supplementary Fig. 1-18

Supplementary Tables 1-3

Supplementary References 1–17

TEM images of In_2O_3 thin film deposited on SiO_2.

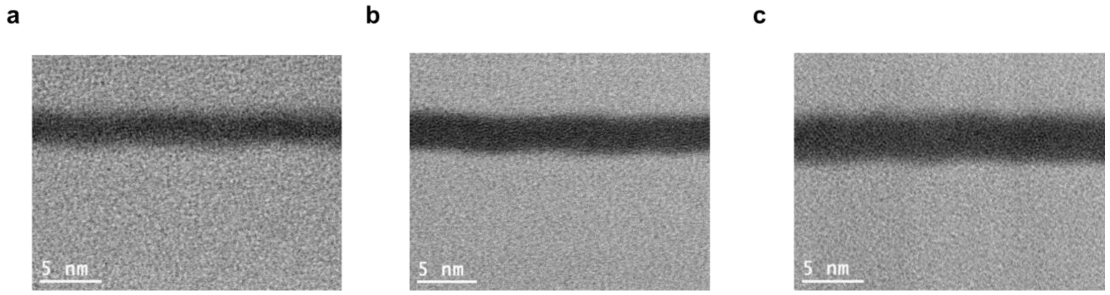

Supplementary Fig. 1 | High-resolution transmission electron microscope (HRTEM) images of In_2O_3 with different thicknesses. a, ~2 nm **b,** ~3 nm **c,** ~4 nm.

AFM images of In_2O_3 thin film deposited on SiO_2.

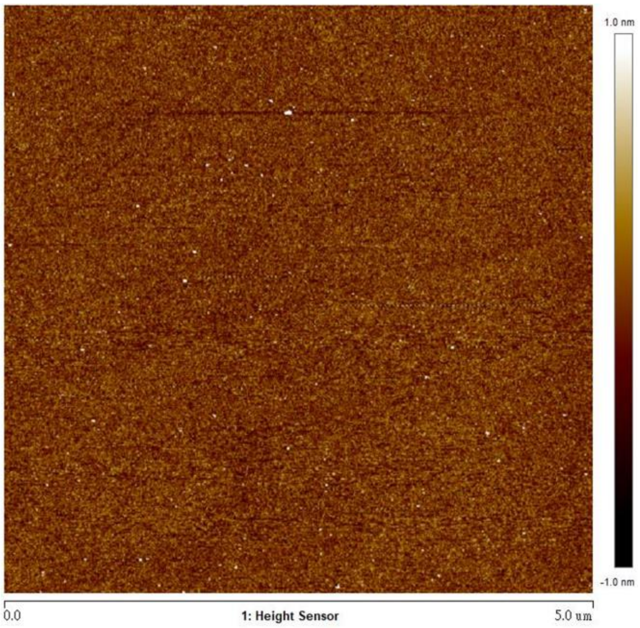

Supplementary Fig. 2 | AFM data with 2 nm In_2O_3. The roughness of R_q = 0.19 nm and R_a = 0.14 nm.

Devices sampling

We fabricate 10 separate batches of devices using an independent and identical process to examine the device-to-device variation. For each batch, we measured 5 devices and gathered a total of 50 independent data.

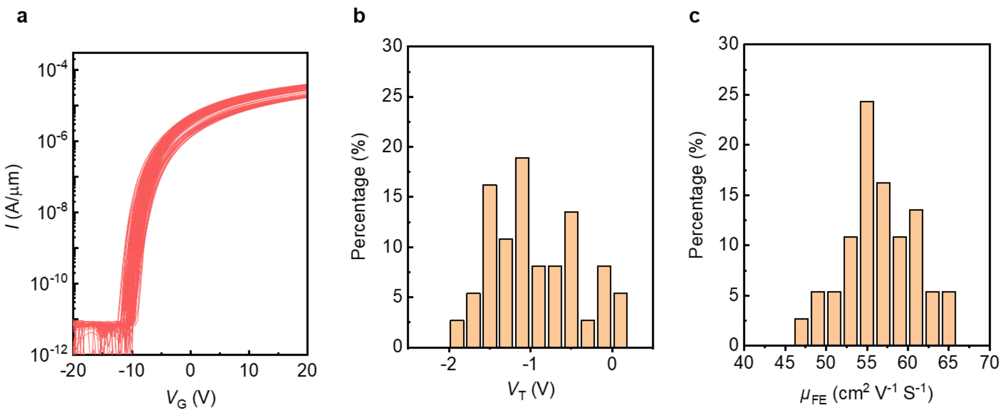

Supplementary Fig. 3 | Devices sampling. a, Transfer curves **b,** Histogram of V_T **c,** Histogram of μ_{FE}.

Indium oxide absorption spectra

Supplementary Fig. 4 shows the absorption spectra of varied thickness In_2O_3. To obtain the bandgap of In_2O_3 with different thickness, the absorption spectra is converted to Tauc Plot to extract the bandgap, as shown in Fig. 1b. The Tauc plot method is based on the assumption that the absorption coefficient α could be expressed by the following formula:

$$(\alpha \cdot h\nu)^{1/\gamma} = A(h\nu - E_g)$$

Where h is the Planck constant, ν is the photon's frequency, E_g is the band gap energy, and A is a constant. The γ factor depends on the nature of the electron transition and is equal to 1/2 or 2 for the direct and indirect transition band gaps, respectively. Here, the γ factor is equal to 1/2 for In_2O_3.

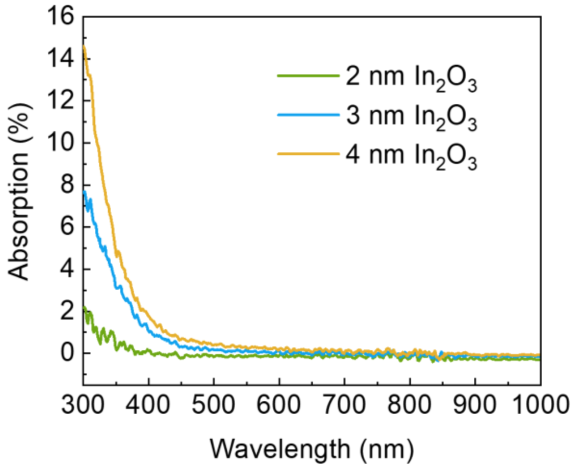

Supplementary Fig. 4 | Thickness-dependent indium oxide absorption spectra.

Characteristics of In_2O_3 transistors below 2 nm

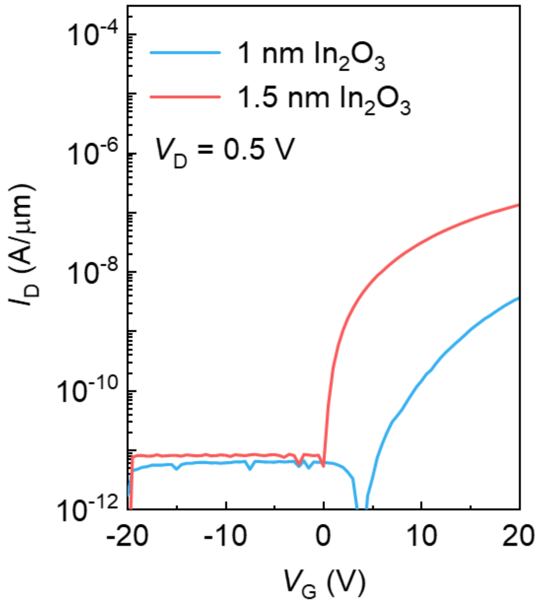

Supplementary Fig. 5 | Devices characteristics with 1/1.5 nm channel thickness and channel width/length of 10/2 μm.

Saturation behavior (I_D-V_D) of 2 nm In_2O_3 transistor

The saturation mobility extracted from $\mu_{sat} = \frac{2 I_D L}{W C_{OX}(V_G - V_T)^2}$ at the saturation regime is 60.9 $cm^2\ V^{-1}\ s^{-1}$

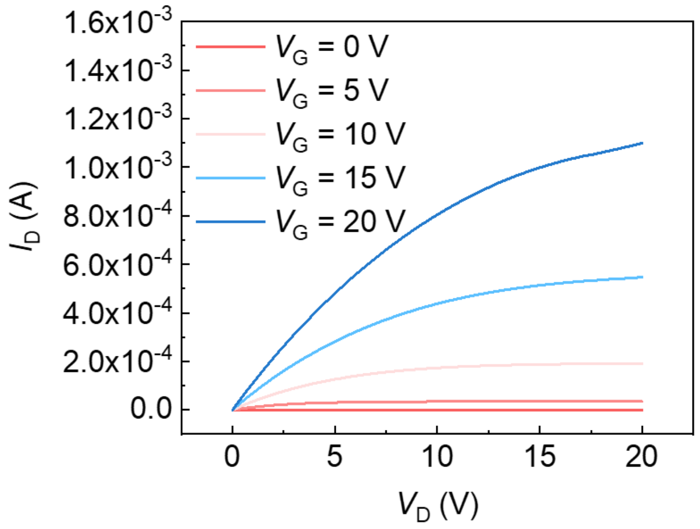

Supplementary Fig. 6 | I_D - V_D characteristics of an In_2O_3 transistor with channel width/length of 10/2 µm.

Characteristics of ultra-thin indium oxide transistor under different wavelength laser illumination

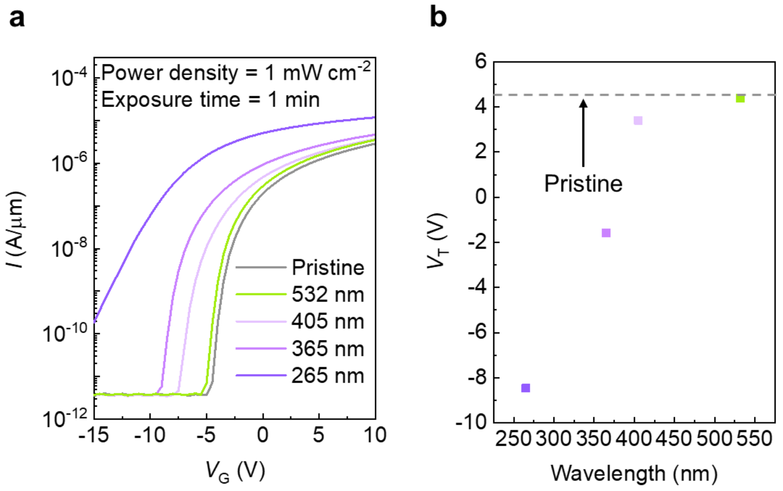

Supplementary Fig. 7 | Characteristics of ultra-thin indium oxide transistor under different wavelength laser illumination. a, The transfer curves of 2 nm In_2O_3 transistor with channel width/length of 10/2 µm under 265, 365, 405, 532 nm laser illumination with the same absorbed light power density 1 mW cm^{-2} for 1 min. **b**, V_T of ultra-thin indium oxide transistor from **a**.

V_T shift with various laser power density for various times.

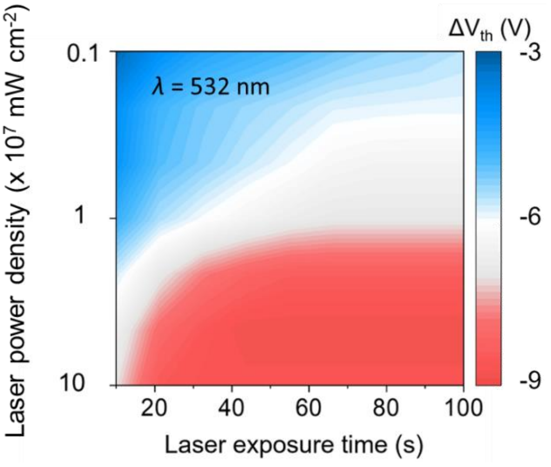

Supplementary Fig. 8 | A contour plot of V_T variation with absorbed power density increasing from 0.1 mW cm^{-2} to 10 mW cm^{-2} for exposure times from 10 s to 100 s under 532 nm laser illumination. Devices were annealed at 150 °C in O_2 for 30 minutes to reset the VT before each UV exposure measurement. The measurement time interval is 10s and the transfer characteristics of the device are immediately measured (< 5 s) after 532 laser illumination. The plot consists of 70 data points. (7 set of different power density and 10 set of time interval)

Theshold voltage step sampling under same illumination condition

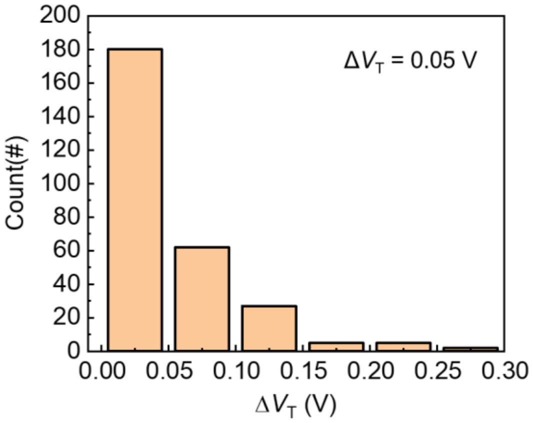

Supplementary Fig. 9 | Histogram of ΔV_T under a power density of 0.1 mW cm^{-2} with the same time period of 1 s for several times.

Transfer curves for different substrate and electrodes

To verify the reproducibility, we tested devices with identical fabrication processes but different metal contacts (Pd, Pt) and substrates (10 nm HfO_2 as the back gate dielectric). All results exhibited consistent trends. The device shows negative V_T shift after UV exposure and positive V_T shift after O_2 annealing, regardless of substrate and metal contacts.

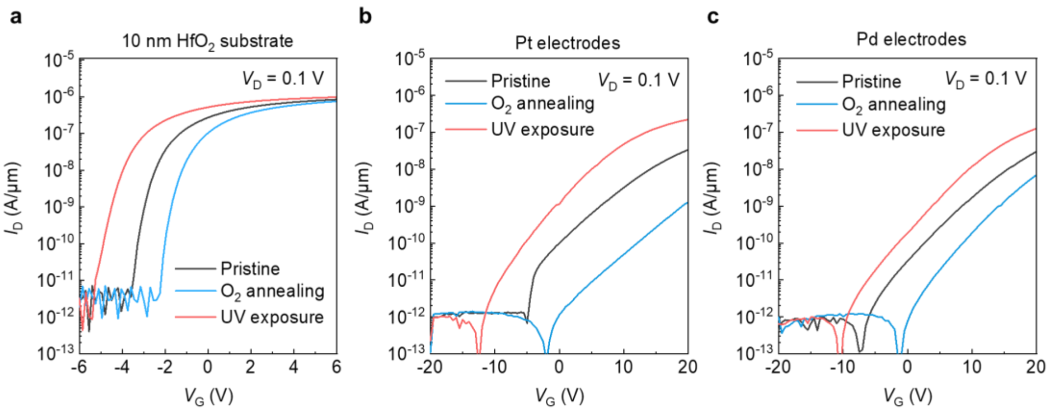

Supplementary Fig. 10 | Transfer curves for different substrate and electrodes with O_2 annealing and UV exposure. a, Transfer characteristics of 2 nm In_2O_3 on ALD 10 nm HfO_2 devices with 40 nm Ni electrodes and channel width/length of 10/2 µm. **b,** Transfer characteristics of 2 nm In_2O_3 on ALD 30 nm SiO_2 devices with 40 nm Pt electrodes and channel width/length of 10/2 µm. **c,** Transfer characteristics of 2 nm In_2O_3 on ALD 30 nm SiO_2 devices with 40 nm Pd electrodes and channel width/length of 10/2 µm.

Bias stress experiment

The results show that the In_2O_3 transistors are subjected to the V_T shifts under the gate bias stresses and the direction of the shift depends on the polarity of the biases. The trends observed in PBS and NBS for pristine, UV-exposed, and O_2-annealed devices are similar, suggesting that these processes minimally affect the physical properties of the channel, primarily altering the carrier concentrations.

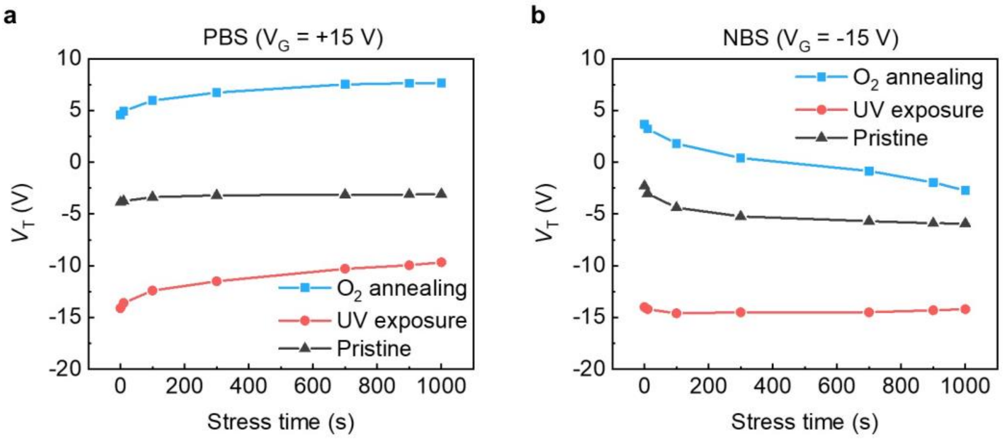

Supplementary Fig. 11 | PBS and NBS performance with pristine, O_2 annealing and UV exposure. a, Positive bias stress (PBS) and **b,** Negative bias stress (NBS) of the In_2O_3 transistors. The bias results are performed with devices with thickness of 2 nm and channel width/length of 10/2 µm.

Temperature simulations with laser exposure

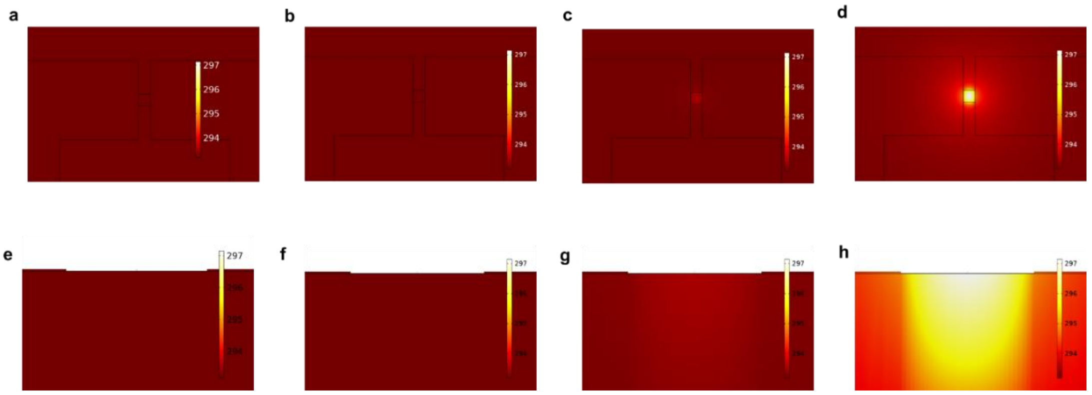

Supplementary Fig. 12 | Temperature variation with different incident UV laser power density. a, A top view of temperature variation with incident power density of 1×10^2 mW cm^{-2}. **b,** A top view of temperature variation with incident power density of 2×10^5 mW. **c,** A top view of temperature variation with incident power density of 2×10^6 mW. **d,** A top view of temperature variation with incident power density of 2×10^7 mW. **e~h,** side view of **Supplementary Fig. 12a~d**. The spot radius of UV laser is 2.54 µm. The active area of channel is 3 µm × 3 µm of 2 nm In_2O_3 on 30 nm SiO_2 with 1 mm ×1 mm Si substrate. The results show that the temperature variation is below 10 °C when exposed to a power density of 2×10^2 mW cm^{-2} which exceeds the power density applied to the devices by five orders of magnitude.

Channel length dependent characteristics

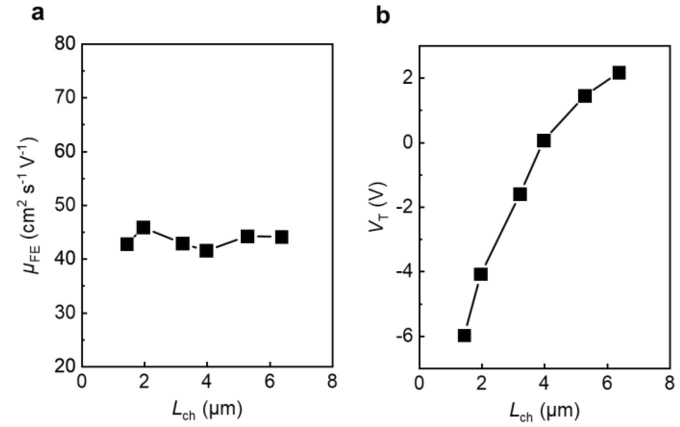

Supplementary Fig. 13 | Devices characteristics with different channel length (channel width = 10 μm). a. μ_{FE} **b.** V_T

Reversibility of V_T tuning *via* thermeral annealing

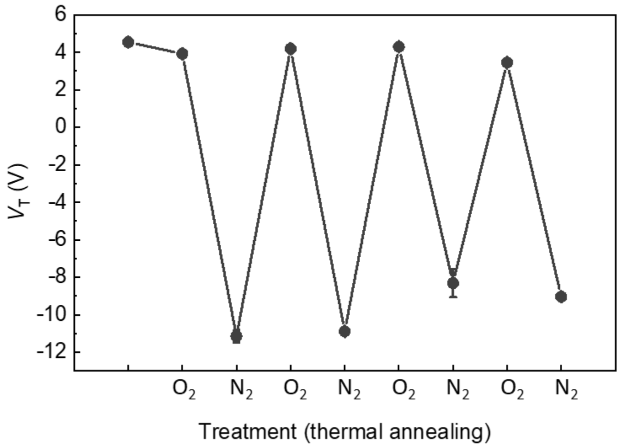

Supplementary Fig. 14 | Reversibility of V_T tuning in ultrathin In_2O_3 transistors. V_T of a 2 nm In_2O_3 transistor with channel width/length of 10/2 μm during multiple O_2 annealing and N_2 annealing cycles. In_2O_3 transistors are thermally annealed under O_2 for 30 minutes at 150°C. Error bars represent 5 individual data.

Scanning tunneling spectroscopy

The scanning tunneling spectroscopy measurement revealed that the bandgap of 2 nm In_2O_3 was around 3 eV, corresponding to the result of the optical bandgap from absorption spectra (Fig. 1b).

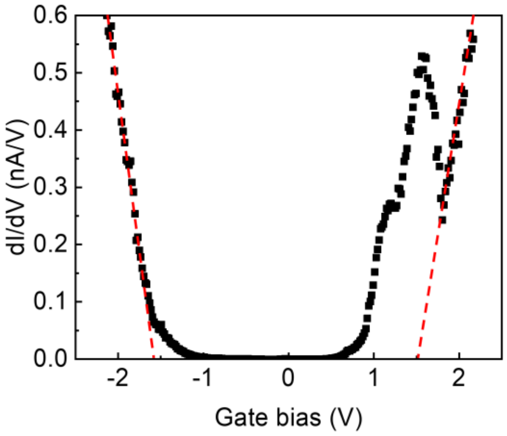

Supplementary Fig. 15 | Scanning tunneling spectroscopy (STS) measurement performed on 2 nm In_2O_3 film.

Air stability of ultra-thin indium oxide transistor

We also measured V_T shift with time as shown in Supplementary Fig. 16. The V_T of In_2O_3 after laser exposure annealing would gradually return to a certain value in a week and saturate finally, which means that the discharged O_2 would absorb back to the surface of In_2O_3 and reach dynamic equilibrium in the air. Nevertheless, we could preserve the devices in a high vacuum system or coating passivation layer on the surface of In_2O_3 to enhance the air stability of ultra-thin In_2O_3 transistors.

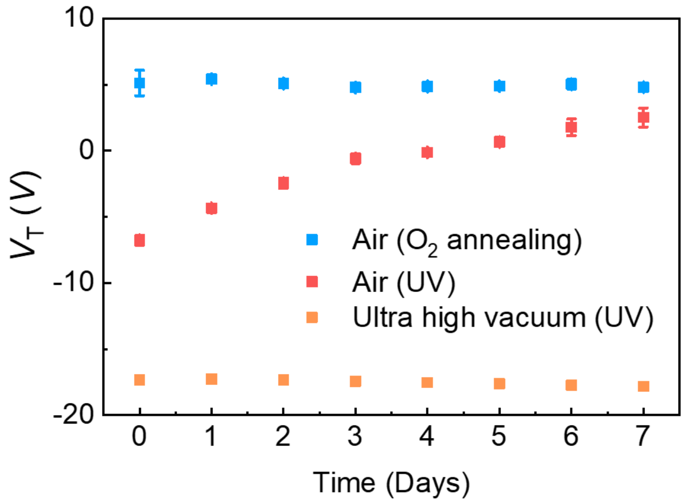

Supplementary Fig. 16 | The V_T shift with time. V_T after O_2 150 °C for 30 min and measured in the air for 7 days (blue line). V_T after 365 nm laser illumination with absorbed laser power density 100 mW cm^{-2} for 1 min and measured in the air for 7 days (red line). V_T after 365 nm laser illumination with absorbed laser power density 100 mW cm^{-2} for 1 min and measured in ultra-high vacuum (~5×10^{-9} torr) for 7 days (yellow line). Error bars represent 5 individual data.

Multi-step transistor

The first increasing part of the current could be referred to the minima length channel, which was illuminated for the longest time to shift V_T negatively. After that, the second channel segment would also be illuminated by laser for a shorter exposure time to make the second state of V_T. Then, the ternary logic function was demonstrated in a single device.

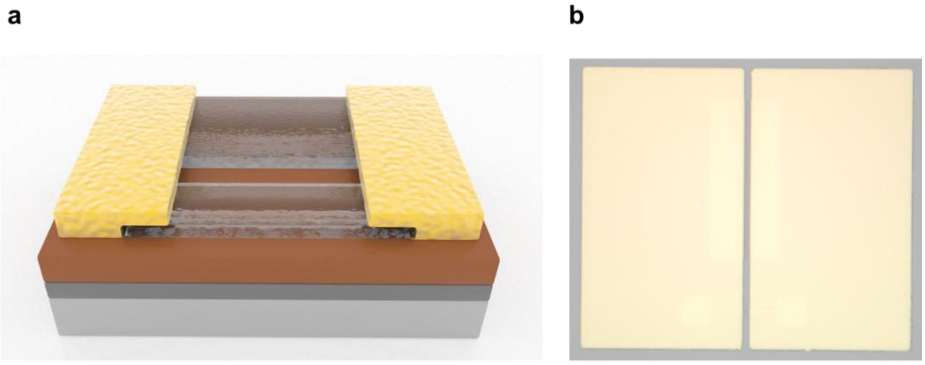

Supplementary Fig. 17 | Device structures of ultra-thin In_2O_3 multi-step transistor. **a,** Three-dimensional schematic view of a multi-step In_2O_3 transistor. **b,** The optical image of a multi-step In_2O_3 transistor.

Neuromorphic Computing

With the ability to fine-tune V_{th} in a wide range under laser illumination, the ultra-thin In_2O_3 transistor shows great potential for optoelectronic synapses. The human brain has been seen as an ultimate computing system, which can compute and store data simultaneously with low energy consumption. The human visual system could transform the light stimulations into electrical pulses and transmit them to the visual cortex. Similarly, the ultra-thin In_2O_3 transistor exhibits high sensitivity to the 365 nm laser illumination and converts light pulses into an electrical signal. Also, the signal could be stored at a different level to realize neuromorphic functions. A typical photo-responsive characteristic of the ultra-thin In_2O_3 transistor is shown in Supplementary Fig. 18a. For the UV laser photo responsivity measurement, the reading bias was set at a fixed value of 0.5 V. The current increased increased from the background current of 180 nA to 690 nA upon 365 nm UV laser illumination at 1 mW cm^{-2} for 150 s, followed by a gradual decay when the light is off. The long decay time constant positions ultra-thin In_2O_3 as an ideal candidate for non-volatile memory applications. Under the pulsed laser stimulation with varied frequencies, the transition process from short-term memory (STM) to long-term memory (LTM) was well demonstrated in an ultra-thin In_2O_3 transistor, as shown in Supplementary Fig. 18b.

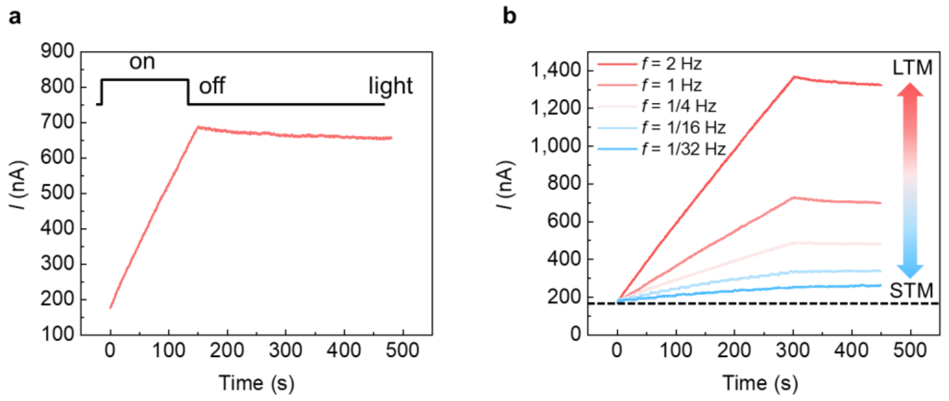

Supplementary Fig. 18 | Optical characteristic of ultra-thin In_2O_3 transistor

a, Typical current evolution process of ultra-thin In_2O_3 transistor under UV laser with an intensity of 1 mW cm^{-2} **b,** Frequency of pulsed light stimulation.

Element scarcity

Element	%	Price (USD/kg)
**O**	46.1	0.154
**Si**	28.2	1.7
**Al**	8.23	1.79
**S**	0.035	0.093
**C**	0.02	0.122
**Zn**	0.007	2.55
**Cu**	0.006	6
**N**	0.0019	0.14
**Ga**	0.0019	148
**Sn**	0.00023	18.7
**As**	0.00018	0.999-1.31
**Ge**	0.00015	914 - 1010
**Mo**	0.00012	40.1
**In**	0.000025	167
**Sb**	0.00002	5.79
**Se**	0.000005	21.4
**Te**	1.00E-07	63.5

Supplementary Table 1 | The abundance and price of common semiconductor element (acquired from wikipedia).

Benchmark of 2D carrier density

Drude model

The 2D carrier density is estimated from $n_{2D} = I_D L/(qWV_D\mu)$, where q is the electron charge, I_D is the source-drain current at zero gate voltage, V_D is the source-drain voltage, and μ is the field-effect carrier mobility.

Parallel-plate capacitor model (ΔV_T)

The 2D carrier density is estimated from $n_{2D} = C_{ox}\,\Delta V_T/q$, where $C_{ox} = \varepsilon_0\varepsilon_r/d$, $\varepsilon_0 = 8.85 \times 10^{-14}$ F/cm, $\varepsilon_r = 3.9$, $d =$ 30 nm (the thickness of SiO_2), $\Delta V_T =$ the change in threshold voltage, q is the elementary charge. The carrier density estimated by the parallel-plate capacitor model means the change in carrier density during tuning process.

Material	Thickness	Dopant	Type	Doping concentration [cm^{-2}]	Model	Refs
MoS_2	3 L	K	n	1.0×10^{13}	Drude	1
	6 L (3.9 nm)	Cl	n	9.2×10^{12}	Drude	2
	7 L (5 nm)	BV	n	1.2×10^{13}	Drude	3
	10 L (7 nm)	$AuCl_3$	p	1.5×10^{12}	Drude	4
	3 L (1.95 nm)	SAMs	n	2.2×10^{12}	ΔV_T	5
	1 L	Pentamethylrhodocene dimer	n	2.4×10^{12}	ΔV_T	6
	1 L	$[N(C_6H_4\text{-p-Br})_3]SbCl_6$	p	6.9×10^{12}	ΔV_T	6
$MoSe_2$	1 L (0.7 nm)	W	p	4.0×10^{11}	Drude	7
	1 L	Pentamethylrhodocene dimer	n	1.7×10^{12}	ΔV_T	6
	1 L	$[N(C_6H_4\text{-p-Br})_3]SbCl_6$	p	9.6×10^{12}	ΔV_T	6
WS_2	5 L (4 nm)	Cl	n	6.0×10^{11}	Drude	2
	1 L (0.84 nm)	N	p	3.8×10^{11}	Drude	8
	1 L	Pentamethylrhodocene dimer	n	2.1×10^{12}	ΔV_T	6
	1L	$[N(C_6H_4\text{-p-Br})_3]SbCl_6$	p	7.5×10^{12}	ΔV_T	6
WSe_2	5 L	K	n	2.5×10^{12}	Drude	1
	1 L (0.7 nm)	NO_2	p	2.2×10^{12}	Drude	9
	5 L (3.8 nm)	PPh_3	n	7.7×10^{11}	ΔV_T	10
	1 L	Pentamethylrhodocene dimer	n	1.1×10^{12}	ΔV_T	6
BP	10 L (8.4 nm)	F4-TCNQ	p	2.2×10^{13}	Drude	11
In_2O_3	~2 nm	-	n	2.8×10^{12}	Drude	This work
	~2 nm	-	n	1.5×10^{13}	ΔV_T	This work
	~3 nm	-	n	2.3×10^{13}	Drude	This work
	~3 nm	-	n	2.3×10^{13}	ΔV_T	This work
	~4 nm	-	n	6.4×10^{13}	Drude	This work
	~4 nm	-	n	2.8×10^{13}	ΔV_T	This work

Supplementary Table 2 | Summary of 2D carrier density with different doping techniques.

Material	Thickness	Inserted layer	Type	Carrier density [cm^{-2}]	Model	Refs
**MoS_2**	1 L (0.65 nm)	P(VDF-TrFE)	Ferroelectric	1.0×10^{12}	ΔV_T	[12]
	3 L (2.1 nm)	PZT	Ferroelectric	7.8×10^{12}	ΔV_T	[13]
**BP**	8 L (7 nm)	BP	Flash	3.0×10^{12}	ΔV_T	[14]
	9 L (8 nm)	MoS_2	Flash	1.0×10^{12}	ΔV_T	[15]
**Graphene**	1L (0.4 nm)	MoS_2	Ferroelectric	1.3×10^{12}	ΔV_T	[16]
	1 L	PMN-PT	Flash	6.7×10^{12}	ΔV_T	[17]

Supplementary Table 3 | Summary of carrier density with carrier density of flash and ferroelectric non-volatile memories.

Supplementary References

1. Fang H, Tosun M, Seol G, Chang TC, Takei K, Guo J*, et al.* Degenerate n-doping of few-layer transition metal dichalcogenides by potassium. *Nano. Lett.* 2013, **13**(5)**:** 1991-1995.
2. Yang L, Majumdar K, Liu H, Du Y, Wu H, Hatzistergos M*, et al.* Chloride molecular doping technique on 2D materials: WS_2 and MoS_2. *Nano. Lett.* 2014, **14**(11)**:** 6275-6280.
3. Kiriya D, Tosun M, Zhao P, Kang JS, Javey A. Air-stable surface charge transfer doping of MoS_2 by benzyl viologen. *J. Am. Chem. Soc.* 2014, **136**(22)**:** 7853-7856.
4. Liu X, Qu D, Ryu J, Ahmed F, Yang Z, Lee D*, et al.* P-type polar transition of chemically doped multilayer MoS_2 transistor. *Adv. Mater.* 2016, **28**(12)**:** 2345-2351.
5. Li Y, Xu C-Y, Hu P, Zhen L. Carrier control of MoS_2 nanoflakes by functional self-assembled monolayers. *ACS Nano* 2013, **7**(9)**:** 7795-7804.
6. Zhang S, Hill HM, Moudgil K, Richter CA, Hight Walker AR, Barlow S*, et al.* Controllable, Wide-Ranging n-Doping and p-Doping of Monolayer Group 6 Transition-Metal Disulfides and Diselenides. *Adv. Mater.* 2018, **30**(36)**:** 1802991.
7. Li X, Lin MW, Basile L, Hus SM, Puretzky AA, Lee J*, et al.* Isoelectronic tungsten doping in monolayer $MoSe_2$ for carrier type modulation. *Adv. Mater.* 2016, **28**(37)**:** 8240-8247.
8. Tang B, Yu ZG, Huang L, Chai J, Wong SL, Deng J*, et al.* Direct n-to p-type channel conversion in monolayer/few-layer WS_2 field-effect transistors by atomic nitrogen treatment. *ACS Nano* 2018, **12**(3)**:** 2506-2513.
9. Fang H, Chuang S, Chang TC, Takei K, Takahashi T, Javey A. High-performance single layered WSe_2 p-FETs with chemically doped contacts. *Nano Lett.* 2012, **12**(7)**:** 3788-3792.
10. Jo SH, Kang DH, Shim J, Jeon J, Jeon MH, Yoo G*, et al.* A High-Performance WSe2/h-BN Photodetector using a Triphenylphosphine (PPh_3)-Based n-Doping Technique. *Adv. Mater.* 2016, **28**(24)**:** 4824-4831.
11. Du Y, Yang L, Zhou H, Peide DY. Performance enhancement of black phosphorus field-effect transistors by chemical doping. *IEEE Electron Device Lett* 2016, **37**(4)**:** 429-432.
12. Lee HS, Min SW, Park MK, Lee YT, Jeon PJ, Kim JH*, et al.* MoS_2 nanosheets for top-gate nonvolatile memory transistor channel. *Small* 2012, **8**(20)**:** 3111-3115.

13. Lu Z, Serrao C, Khan AI, Clarkson JD, Wong JC, Ramesh R*, et al.* Electrically induced, non-volatile, metal insulator transition in a ferroelectric-controlled MoS_2 transistor. *Appl. Phys. Lett.* 2018, **112**(4)**:** 043107.
14. Lee YT, Lee J, Ju H, Lim JA, Yi Y, Choi WK*, et al.* Nonvolatile charge injection memory based on black phosphorous 2D nanosheets for charge trapping and active channel layers. *Adv. Funct. Mater.* 2016, **26**(31)**:** 5701-5707.
15. Li D, Wang X, Zhang Q, Zou L, Xu X, Zhang Z. Nonvolatile floating-gate memories based on stacked black phosphorus–boron nitride–MoS_2 heterostructures. *Adv. Funct. Mater.* 2015, **25**(47)**:** 7360-7365.
16. Sup Choi M, Lee G-H, Yu Y-J, Lee D-Y, Hwan Lee S, Kim P*, et al.* Controlled charge trapping by molybdenum disulphide and graphene in ultrathin heterostructured memory devices. *Nat. Commun.* 2013, **4**(1)**:** 1-7.
17. Jie W, Hui YY, Chan NY, Zhang Y, Lau SP, Hao J. Ferroelectric polarization effects on the transport properties of graphene/PMN-PT field effect transistors. *J. Phys. Chem. C* 2013, **117**(26)**:** 13747-13752.
