# Supplementary material for: Wide-range and area-selective threshold voltage tunability in ultrathin indium oxide transistors
Source: Nat Commun. 2023 Aug 28;14:5243. doi: 10.1038/s41467-023-41041-y (PMC10462674; doi:10.1038/s41467-023-41041-y)
Supplement: Supplementary file 3 — Description of Additional Supplementary Files [file 41467_2023_41041_MOESM3_ESM.pdf]

Description of Additional Supplementary Files

File Name: Supplementary Movie 1

Description: Automated laser illumination system. The movie shows a process where lasers are fixed while films are held on a moving platform. This setup enables area-selective laser exposure with high throughput.
